# Supplementary material for: Carbon fixation from mineral carbonates
Source: Nat Commun. 2017 Oct 18;8:1025. doi: 10.1038/s41467-017-00703-4 (PMC5715163; doi:10.1038/s41467-017-00703-4)
Supplement: Supplementary file 1 — Supplementary Information [file 41467_2017_703_MOESM1_ESM.pdf]

Description of Supplementary Files

File Name: Supplementary Information
Description: Supplementary Figures and Supplementary Table

File Name: Peer Review File
Description:

^{13}C analysis of DIC post culture incubation in tracer medium ($\delta^{13}C$ = 5479 ‰)

Inoculum	Calcite chips	$\delta^{13}C$ (‰)	SD*
None	No	5072.8	4.9
None	Yes	5053.9	28.4
**Boring**	**Yes**	**1778.2**	**3.7**
Planktonic	No	4089.3	6.7

* Standard deviations are derived from instrumental technical replication of each gas injection.

Supplementary Table 1

A culture containing a boring inoculum (Shaded), and showing abundant endolithic growth showed a final medium $\delta^{13}C$ value of only 1778.2 ± 3.7‰, indicating significant dilution (to 32% of initial tracer) with a lighter source. A control culture using only planktonic inoculum and no mineral to bore, showed only a minimal dilution, probably due to respired organic C from the inoculum itself (final C_i $\delta^{13}C$ of 4089.3 ± 6.7‰.). Thus, a significant dilution with light carbon, which could only come from the mineral, occurred only in the presence of actively boring biomass within a mineral substrate, which is consistent with our initial hypothesis. In terms of mass balance, because the cultures contained 0.02 mmol DIC per vial initially, a tracer dilution to 0.32% of the initial in the boring incubation implies that at least 0.04 mmol (2 mg) of calcite was biologically dissolved, corresponding to a solid volume of 0.74 mm^3, which is roughly 3% of the calcite volume available. This calculation does not include the calcite C converted into biomass, which can be approximated noting that a unit volume of calcite excavated, when replaced by the same volume of canonical microbial biomass will contain about 1/3 of the C content. Hence the total excavation in the experiments can be deemed to be some 5% of calcite volume available. These data align well with the notion that significant excess C_i is released during the boring process, a result also consistent with the pattern in natural abundance $\delta^{13}C$ of the planktonic, benthic, and endolithic *M. testarum* biomass (Fig. 2A). This has, of course, ecological implications in that the release of mineral-derived C can subsequently be fixed by epilithic or benthic biomass. It is thus feasible that excess C_i liberated by euendolithic biomass during carbonate mineral excavation could suffice to sustain epilithic photosynthetic biomass. In this way, and through grazing by benthic herbivores, like chiton or other mollusks, the influence (and isotopic signature) of mineral C_i sources may extend beyond the lithic environment itself.

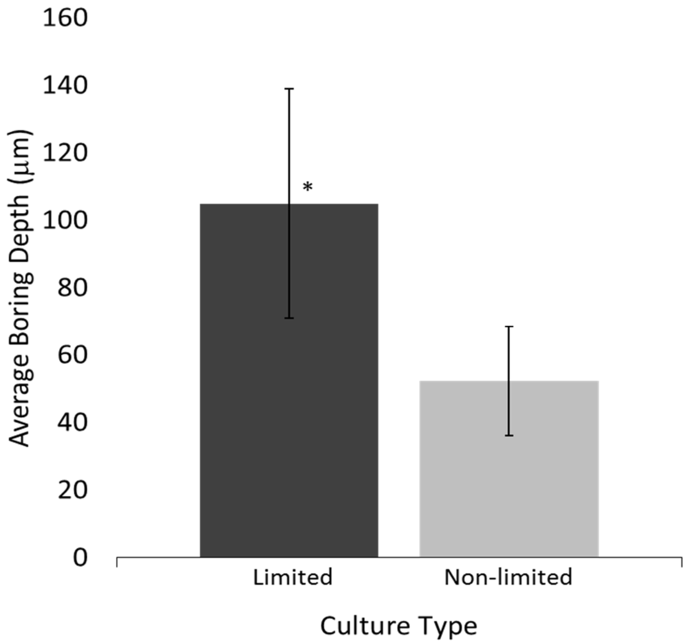

Supplementary Fig. 1 Maximal boring bed penetration depth within chips grown under C_i limited and non-limited cultures after a 4-month incubation (n=6). All chips were from cultures started with endolithic inoculum. Means were significantly different (p<0.01, ANOVA). Error bars represent one standard deviation.

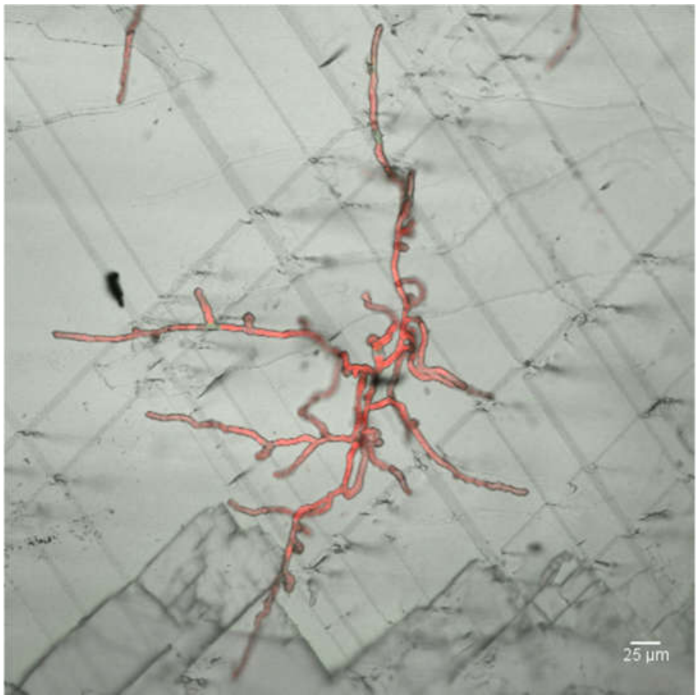

Supplementary Fig. 2 A single boring radial colony of *M. testarum* grown inside of a crystalline calcite chip after a 3 weeks of inoculation with sheared planktonic biomass. Colonies such as this one constituted the basis for the 'endolithic inoculum' used in the experiments. Photomicrograph is an overlay of a (gray scale) differential interference contrast image, with a confocal fluorescent image (red) based on photopigment autofluorescence (excitation at 561 nm, emission at 660-700 nm) all taken on a Leica SP5 LASER Confocal Microscope.

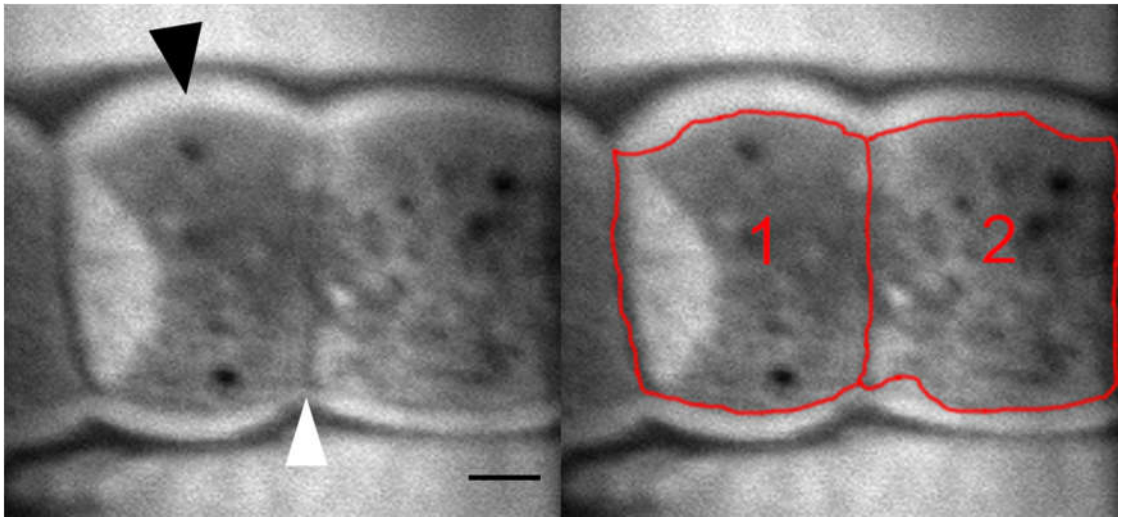

Supplementary Fig. 3 An example of a single NanoSIMS image with (right) and without (left) regions of interests (ROI) drawn to indicate cells used to extract isotopic data. The sheath (when observable, black arrowhead) was excluded from analysis and individual cells segmented using the visible cell septum (white arrowhead). This is the same field of view shown in Fig. 2-B Outside. Scale bar is 2 microns.
